# More than Just Pneumonia: Acute Pulmonary Embolism in Two Middle-Aged Patients with COVID-19

**DOI:** 10.1155/2020/4812036

**Published:** 2020-07-30

**Authors:** Jahinover Mazo, Sukhdev Singh, Zohaib Khan, Allison Foster, Ecaterina Komarnitsky, Abhiram Nagaraj, Soham Patel, Vinaya Kikkeri

**Affiliations:** ^1^Department of Radiology, Richmond University Medical Center, New York, NY, USA; ^2^College of Medicine, American University of Antigua, New York, NY, USA

## Abstract

**Background:**

Although severe pneumonia and respiratory compromise have remained the predominant complications of coronavirus disease 19, we are now learning this virus is much more varied in its presentation. In particular, there are increasingly reported cases of thromboembolic events occurring in infected patients. *Case Report*. In this report, we present two patients, both under the age of 40 with known risk factors for venous thromboembolism, who presented with respiratory distress. Both patients were diagnosed with SARS-CoV-2 pneumonia and pulmonary embolism requiring management with anticoagulation. Both patients were discharged after a short course in the hospital.

**Conclusion:**

The discussion of a hypercoagulable state induced by coronavirus disease 19 has been well documented; however, the exact mechanisms remain unknown. We suspect that a prothrombotic inflammatory response provoked by coronavirus disease could be the culprit, acting as an additive effect on middle-aged patients with known risk factors for venous thromboembolism. We recommend clinicians closely monitor those with known risk factors for pulmonary embolism.

## 1. Introduction

The outbreak of coronavirus disease 19 (COVID-19), caused by SARS-CoV-2, rapidly spread globally in early 2020. As of May 16, 2020, the World Health Organization has reported COVID-19 has been responsible for over 300,000 deaths with over 4.4 million confirmed infections worldwide. According to data from China, where the virus originated, 87% of COVID-19 patients were between the age 30–79 years [1]. The most common symptoms amongst those infected were fever, cough, fatigue, muscle pain, diarrhea, and pneumonia [2]. The most critical cases progressed to respiratory failure, septic shock, and multiorgan failure [2].

An often-unrecognized complication of COVID-19 pneumonia is pulmonary embolism (PE). Recent studies indicate that PE in COVID-19 patients had a prevalence of 23% [3]. Even with DVT prophylaxis and initial improvement, the risk of PE remains high [4]. Lab findings of elevated D-dimer, thrombocytopenia, and increased coagulation parameters are associated with worse outcomes [4]. Interestingly, autopsy findings of COVID-19 patients have shown evidence of pulmonary microthrombi and pulmonary hemorrhage that suggest hypercoagulability may be a key but infrequently reported feature of this disease [5]. We further suspect that middle-aged patients with known risk factors for venous thromboembolism are at an even higher risk due to COVID-19.

## 2. Case Report

### 2.1. Case 1

A 38-year-old obese male presented to the emergency department (ED) for worsening dyspnea and palpitations over the past three days. Earlier in the day, he described feeling like his heart was “jumping out from his chest.” His occupation requires him to sit for long periods and he endorsed that for the past week, he had progressively worsening pain in his legs. He is a former smoker; however, he quit three years ago.

Vitals on presentation were a temperature of 36.8°C, blood pressure of 80/60 mmHg, pulse rate of 148 beats/minute, respiratory rate of 26 breaths/minute, and oxygen saturation of 83% on ambient air which improved to 97% on a non-rebreather mask. Chest auscultation was notable for bilateral crackles, wheeze, and stridor.

**Figure 1:**
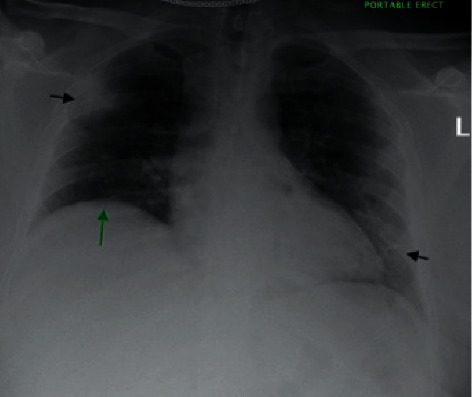
A portable chest radiograph on day 1 of presentation shows bilateral patchy infiltrates (black arrows) in the right upper lung and left lower lung, with mild elevation of the right hemidiaphragm (green arrows).

**Figure 2:**
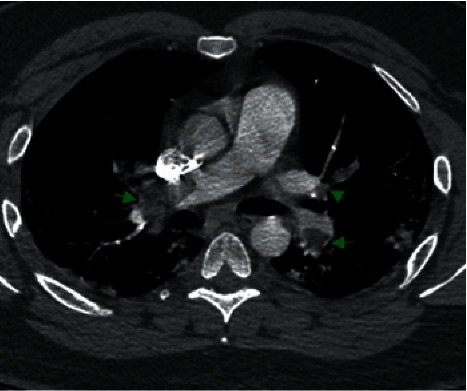
Chest axial CT image on a vascular window shows extensive bilateral multifocal acute pulmonary embolism (green arrows) without any radiological evidence of right heart strain.

**Figure 3:**
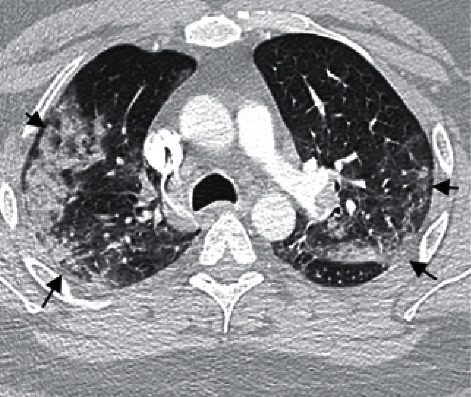
Chest axial CT image on a lung window shows extensive patchy solid and ground-glass opacities scattered throughout the lungs and a small consolidation at the left lower lobe, in keeping with advanced multifocal pneumonia (black arrows).

Laboratory abnormalities included thrombocytopenia (117 k/*μ*l), elevated D-Dimer (20.0 *μ*g/ml), elevated aPTT (151.9 s), elevated lactate dehydrogenase (463 U/L), and a positive RT-PCR for SARS-CoV-2. EKG showed sinus tachycardia with rightward axis deviation. Chest X-ray (see Figure 1) demonstrated patchy infiltrates peripherally in the right upper lung and left mid to lower lung with mild elevation of right hemidiaphragm. Computed tomography angiography (CTA) done a few hours later (see Figures 2 and 3) showed extensive prominent multifocal acute bilateral pulmonary embolism and findings consistent with viral pneumonia.

He was given heparin IV 5,000 units in the ED as prophylaxis for DVT as per hospital protocol; however, he was subsequently switched to a one-time subcutaneous injection of enoxaparin 100 mg. Enoxaparin was held as the patient's aPTT levels were noted to be elevated. However, on day three, he was started on the oral anticoagulant, apixaban 10 mg twice daily. After a seven-day hospitalization, he was clinically stabilized and discharged on home oxygen along with instructions to continue oral anticoagulation for three months and follow up as an outpatient.

### 2.2. Case 2

A 39-year-old female with a history of hypertension was sent to the ED from an urgent care facility after a chest X-ray revealed worsening bilateral infiltrates. She complained of progressively worsening left-sided chest and flank pain for three days. The pain was exacerbated by deep inspiration and associated with severe shortness of breath. She was presumed to be SARS-CoV-2 positive by her primary care physician due to her husband's recent positive test. Associated symptoms included cough, fever, sore throat, diarrhea, and body aches for 2–3 weeks. She was prescribed a 12-day course of azithromycin and a five-day course of hydroxychloroquine. On follow-up, the patient's repeat chest x-ray showed new infiltrates, and she was started on a seven-day course of levofloxacin. Although most of the COVID-19 related symptoms subsided with treatment, the patient endorsed mild intermittent hemoptysis. The patient's history also revealed the use of oral contraceptives since 2017.

**Figure 4:**
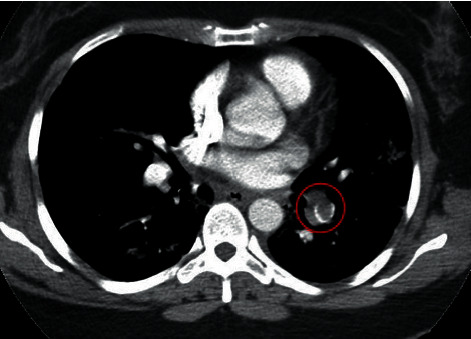
Chest axial CTA image on a vascular window shows a filling defect (circle) in the left lower lobe consistent with lobar and segmental pulmonary embolism.

**Figure 5:**
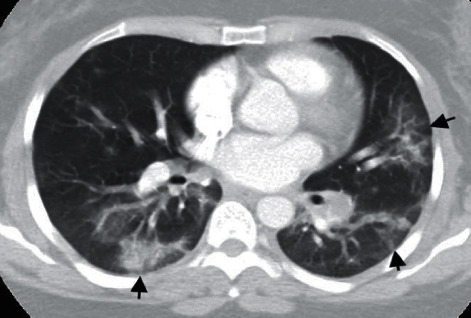
Chest axial CTA image on a lung window shows bilateral multifocal peripheral, lower zonal predominant ground-glass opacities to consolidations (black arrows).

Vitals on presentation were a temperature of 37.2°C, blood pressure of 119/77 mmHg, pulse rate of 110 beats/minute, respiratory rate of 18 breaths/minute, and oxygen saturation of 97% on ambient air. Physical examination was notable for left-sided chest wall tenderness extending from midaxillary line to the scapular region. Labs were notable for hemoglobinemia (10.8 g/dL), lymphopenia (0.9 k/*μ*L), and reactive thrombocytosis (560 k/*μ*L). RT-PCR for SARS-CoV-2 was positive. EKG demonstrated normal sinus rhythm with no ischemia or right ventricle failure. CTA (see Figures 4 and 5) demonstrated a filling defect in the left lower lobe consistent with lobar and segmental pulmonary embolism and findings consistent with the known history of pneumonia.

During hospital stay, she was started on IV heparin 5,000 units as per hospital protocol for DVT prophylaxis and was consequently admitted to the general medical floor for management. During day two of hospital stay, she was switched from IV heparin to subcutaneous enoxaparin 70 mg twice daily for treatment of PE. After a three-day hospital course, she was discharged with instructions to start and continue apixaban for two months and follow up as an outpatient.

## 3. Discussion

Reports of COVID-19 patients presenting with thromboembolic events have been steadily increasing as the pandemic continues. In a recent study of patients with confirmed COVID-19 pneumonia, thrombotic events were reported to occur in 31% of patients, despite appropriate thromboprophylaxis [6]. 81% of these were confirmed cases of acute pulmonary embolism [6]. In another study examining 388 COVID-19 patients with diagnosed thromboembolic events, half occurred within twenty-four hours of hospital admission [7]. While the explanation of the observed phenomena eludes us, evidence suggests a combination of prothrombotic and proinflammatory mediators may hold some answers.

### 3.1. Coagulation Factor Abnormalities

Supporting evidence of an existing hypercoagulable state has been demonstrated by observed increases in prothrombotic factors. Factor VIII, which plays a key role in the coagulation cascade during homeostasis, is known to be bound to von Willebrand factor (vWF) in circulation. Activation of factor VIII leads to a trickling effect, resulting in thrombin activation and thromboembolism formation. Factor VIII was shown to be elevated in patients with COVID-19 associated thrombotic events, whereas derangements of anticoagulant factors were not commonly seen [8]. In addition to coagulation factor abnormalities, other reported findings of increased D-dimers, ferritin, and lactate dehydrogenase further support the assertion that a prothrombotic response to the virus is driving the thromboembolic events among COVID-19 patients [9]. Supplementary studies have demonstrated elevations in partial thromboplastin, prothrombin times, fibrinogen, and D-dimer levels, suggesting widespread activation of the clotting cascade [10]. Additionally, factors such as fibrinogen resulting in increased D-dimers have also correlated with higher mortality [11]. In a study of sixteen COVID-19 patients with thromboembolic complications, the following striking lab values were discovered: elevated clot strength, elevated D-dimer, hyperfibrinogenemia, and elevated IL-6 levels [12].

There have also been reports of patients who suffered strokes despite having sufficient anticoagulation in therapeutic ranges [9]. In these patients, D-dimer elevations were nearly eight times greater than the median of normal reported for COVID-19 patients [9].

### 3.2. Inflammatory Mediators and Hypercoagulability

Severe COVID-19 infections have also been associated with an inflammatory prothrombotic state, also potentially playing a key role behind the increase in reported thromboembolic complications. Elevations in inflammatory mediators in infected patients include IL-6, IL-1, and tumor necrosis factor-*α* [11, 13]. These proinflammatory cytokines, associated with the severity of infection, can injure the endothelium and activate mononuclear cells, leading to tissue factor expression which, in turn, activates the coagulation cascade [9].

TNF and IL-1 are known to exert an antifibrinolytic effect by stimulating the production of plasminogen activator inhibitor-1, thus promoting a prothrombotic state [14]. These cytokines are also responsible for stimulating the release of vWF multimers and increasing the production of factor VII and tissue factor [13]. Furthermore, damage to vessels releases vWF, which demonstrates evidence of endothelial injury in patients with proven thrombotic events [8]. The final product of the coagulation cascade is the formation of thrombin, which, in the case of severe inflammation, overwhelms the body's natural anticoagulants resulting in platelet activation leading to thromboembolism and ultimately pulmonary embolism [9].

In both cases presented, our patients had existing risk factors for venous thromboembolism. In case 1, there was a history of smoking, obesity, and prolonged periods of stasis. In case 2, long-term oral contraceptive use was noted. These risk factors have been well studied and reported in the literature as precipitants of a hypercoagulable state. We suspect that the existing risk factors present along with the superimposed prothrombotic state induced by COVID-19 induced inflammatory response may have precipitated the development of the venous thromboembolism resulting in PE.

### 3.3. Management of Acute VTE

The CHEST Guideline and Expert Panel Report on management of venous thromboembolism (VTE) in COVID-19 patients outlines various recommendations for management of acute VTE. The guidelines recommend that hospitalized COVID-19 patients who develop DVT or PE should receive anticoagulation with LMWH (low-molecular-weight heparin) or IV unfractionated heparin (UFH) [15]. LMWH or fondaparinux is preferred over UFH in critically ill patients. Direct oral anticoagulants (DOAC) are recommended for outpatient management of COVID-19 patients with acute DVT/PE [15]. The recommended duration of anticoagulation treatment is three months [15]. For patients with VTE recurrence despite proper anticoagulation, LMWH adjusted for weight is the current recommendation. Dosage increases of LMWH by 25–50% is advised in refractory cases [15].

At present, the efficacy of thrombolytic therapy in hemodynamically stable COVID-19 patients is not well established and requires further investigation. Therefore, thrombolytics should only be considered in patients with acute ischemic stroke, myocardial infarction, massive PE, and hemodynamic instability [16].

### 3.4. VTE Prophylaxis

Hospitalized patients with COVID-19 can be assessed for risk of developing venous thromboembolism using an appropriate scoring system for critically ill patients. Various scores exist including the IMPROVE, Padua, and Caprini Prediction Scores. International Medical Prevention of Venous Thromboembolism (IMPROVE) estimates the three-month risk of developing venous thromboembolism in hospitalized patients [17]. The Padua Prediction Score is used to determine the risk of developing a venous thromboembolism and when to initiate thromboprophylaxis to minimize the impending outcome [18]. Caprini categorizes patients into four categories including low risk, moderate risk, high risk, and highest risk based on various criteria [19]. Each of these scoring systems has various criteria, and all include a prior history of venous thromboembolism, active or prior cancer, thrombophilia, and immobilization [17–19]. Although higher scores for each risk assessment model demonstrate an increased likelihood of the patient developing a venous thromboembolism and warrant the initiation of thromboprophylaxis, many clinicians have advocated for prophylactic thromboprophylaxis in COVID-19 patients regardless of projected risk.

It is essential to use a patient-specific approach and evaluate any underlying risk factors for VTE which include history of previous VTE, active cancer, immobility, or thrombophilia [16]. Those with contraindications to medication should be placed on compression devices and regularly monitored [20]. For critically ill patients, both pharmacologic and mechanical VTE prophylaxis is recommended [20].

Dosing requirements should be tailored to the patient group. The antithrombotic practice guidelines on thromboprophylaxis for non-ICU hospitalized COVID-19 patients recommend subcutaneous unfractionated heparin twice- or thrice-daily, or once-daily subcutaneous LMWH or fondaparinux [16]. In acutely ill patients, the CHEST guidelines recommend standard dose of anticoagulation with a preference for LMWH or fondaparinux over UFH [15]. For critically ill patients, LMWH is the preferred agent. Obi et al. and their ad hoc committee recommend thromboprophylaxis that consists of LMWH at a dose of 40 mg daily or 30 mg twice daily, or subcutaneous heparin at a dose of 5,000 units three times daily [21].

Critically ill COVID-19 patients should receive the standard dose thromboprophylaxis with a preference for LMWH [15]. Patients with confirmed or high suspicion of thromboembolic disease should receive higher doses of anticoagulation [20]. With improvement, the dose can be reduced to standard VTE once the patient has been downgraded from the ICU to the general medical floor [20].

Following discharge, some COVID-19 patients may require VTE prophylaxis. A multidisciplinary approach involving the patient and health care team should be implemented [20]. Some of the indications for outpatient VTE prophylaxis include patients who required ICU management, were mechanically ventilated, and were paralyzed for a prolonged period or those with VTE risk factors identified on discharge [16, 20]. Suggested regimens include enoxaparin (6–14 day course), rivaroxaban (31–39 day course), and betrixaban (35–42 day course) [20].

## 4. Conclusion

The derangements of coagulation parameters and systemic inflammation coupled with pre-existing risk factors provide the perfect storm for the development of thromboembolic disease. It is imperative to monitor middle-aged patients who have known risk factors for hypercoagulability closely, take proper precautions, and be prepared for such events.
